# Use of traditional and complementary medicine for COVID 19 prophylaxis among healthcare professionals and students in Jordan: A cross-sectional study

**DOI:** 10.1371/journal.pone.0276015

**Published:** 2022-10-20

**Authors:** Nailya Bulatova, Sara Younes, Majd Arabiyat, Ahmad Abukaff, Sara Madanat, Eman Alqudah, Anoud Hamati, Farah Halawa, Abdallah Younes

**Affiliations:** 1 Department of Biopharmaceutics and Clinical Pharmacy, School of Pharmacy, The University of Jordan, Amman, Jordan; 2 Department of Medicine, School of Medicine, The University of Jordan, Amman, Jordan; 3 Private Clinic, Amman, Jordan; Lahore College for Women University, PAKISTAN

## Abstract

**Introduction:**

There is inadequate evidence to recommend the use of any traditional and complementary medicine (T&CM) methods such as vitamin, mineral, herbal or other dietary supplements to prevent or treat COVID 19. Members of the medical team are particularly at risk of exposure to high viral load of coronavirus. They have also the best access to professional information regarding disease treatment and prophylaxis and disseminate such knowledge.

The aim of the study was to assess the prevalence of use of T&CM for the prophylaxis of COVID 19 among the healthcare professionals and students in Jordan, along with the most common types and the factors associated with T&CM use.

**Methodology:**

A cross-sectional study of T&CM use was conducted in Jordan using a snowball sampling method to distribute Google Forms and to enrol participants during coronavirus outbreak between June 10, 2021, and August 28, 2021. The study included healthcare professionals or students who consented to participate in the survey. The survey excluded those participants who had filled the questionnaire at least once or were pregnant/breast-feeding at the time of the study. The questionnaire consisted of 29 items, including screening, checkbox, dichotomous, matrix and open-ended questions.

**Results:**

The response rate was 97.1%. Out of 560 study respondents, 359 (64.1%) reported using T&CM for COVID 19 prevention. Vitamins and nutrients were consumed by almost half (48.4%) of study participants, while nonpharmacological methods and herbal remedies were consumed by 35.2% and 25.2%, respectively. The most common source of information regarding T&CM use for COVID 19 prophylaxis included scientific publications (59.5%), followed by disease treatment guidelines (38.0%) and social media (32.3%). Adverse effects were reported by 8.5% and possible adverse effects were reported by another 8.5% of participants. The T&CM use was associated with working in contact with COVID 19 patients (OR: 1.625 (95% CI 1.047–2.523) (P = 0.03) and having a colleague as a source of information (OR: 1.720 (95% CI 1.026–2.883) (P = 0.04).

**Conclusions:**

The prevalence of T&CM use for COVID 19 prevention among healthcare professionals and students in Jordan is high, with a significant proportion of participants reporting adverse effects. There is an urgent need for further research toward efficacy and safety of T&CM in COVID 19 prophylaxis as well as development of appropriate public health policy on this issue specific to each country.

## Introduction

The novel coronavirus disease 2019 (COVID 19) also named severe acute respiratory syndrome (SARS-CoV-2) is an infectious illness caused by coronavirus. It originated in Wuhan, China, and was initially discovered in December 2019, resulting in a global pandemic [1]. Coronaviruses are positive-stranded RNA viruses with a nucleocapsid and envelope [2]. SARS-CoV-2 demonstrates sustained person-to-person transmission through the air (as respiratory droplets and/ or aerosols) and the direct contact. The median incubation period of the infection is approximately 4−5 days, up to 14 days. Most of COVID-19 patients present with mild to moderate symptoms (81%) [3] including cough, fever, weariness, myalgia, and diarrhea; in severe cases (approximately 14% of infected patients) the disease may cause lung damage [4] and may require ventilation in an intensive care unit (ICU), and 5% eventually develop more critical manifestations such as acute respiratory distress syndrome (ARDS), septic shock, and multiple organ dysfunction or failure [3]. COVID 19 has an impact on many elements of a patient’s life and can be particularly harmful to people with pre-existing health problems [4].

According to the World Health Organization (WHO), as of July 8, 2022, the total cumulative number of cases of COVID 19 worldwide was more than 558 million and total cumulative number of deaths from the infection was more than 6.3 million. In Jordan, the total cumulative number of cases was 1.7 million including 14 068 deaths. Although the situation in many regions of the world has improved due to vaccination and introduction of new therapies, there is more than 1 million of new cases daily, including 1519 deaths [5].

The SARS-CoV-2 virus’ main determinant of viral entry in host cells is the spike (S) glycoprotein, which forms trimers on the surface of virions [6]. To promote viral entry, proteases, including TMPRSS2, furin, and CatB/L, cleave the S protein, with subsequent entry of SARS-CoV-2 and fusion of the viral envelope and endosome, followed by release of the viral ribonucleoprotein complex into the cell [3,6]. The main cells infected by SARS-CoV-2 are nasopharyneal or tracheal mucosal cells. Pattern recognition receptors (PRRs), including the endosomal toll-like receptors (TLR), can detect viral genomic RNA, thus triggering an inflammatory response with involvement of interferons and chemokines. If the virus is not cleared by innate or adaptive immune mechanisms, it can descend from the upper respiratory tract to the lower respiratory tract [6].

In severe COVID-19 cases there is hypoxaemia which can further progress into respiratory failure or acute respiratory distress syndrome (ARDS), a form of lung injury caused by inflammation, pulmonary vascular leakage, and subsequent loss of alveolar tissue. In severe disease, the absence of a proper antiviral response mediated by IFNs has been detected, where the host will rely on other innate immune mechanisms for defence via the production of cytokines and chemokines. CCL2, CCL3, CCL7 and CXCL10 are potent chemokines for monocytes found at high concentrations in patients with severe COVID 19. Increased levels of several cytokines (interleukin (IL)-1β, IL-2, IL-6, IL-7, IL-8, IL-10, IL-17, IFN-γ, IFN-γ-inducible protein 10, MCP-1, G-CSF, MIP-1α, and TNF-α) that leads to systemic inflammatory response (cytokine storm) have been reported in patients with severe COVID-19 [3]. Severe COVID-19 may also lead to extrapulmonary disease, including gastrointestinal symptoms and acute cardiac, kidney and liver injury, in addition to cardiac arrhythmias, rhabdomyolysis, coagulopathy and shock; in extreme cases manifested as multiorgan injury [3,6]. The mechanisms of multiorgan injury include endothelial cell damage, thromboinflammation, dysregulation of the renin−angiotensin−aldosterone system (RAAS) and of the immune response, and direct viral toxicity [3].

The WHO gave their definition of traditional and complementary medicine (T&CM) as follows: “Traditional medicine…is the sum total of the knowledge, skill, and practices based on the theories, beliefs, and experiences indigenous to different cultures, whether explicable or not, used in the maintenance of health as well as in the prevention, diagnosis, improvement, or treatment of physical and mental illness”, while the complementary (alternative) medicine (CAM) is defined as “a broad set of health care practices that are not part of that country’s own tradition or conventional medicine and aren’t completely incorporated into the main health-care system. They are used interchangeably with traditional medicine in some countries” [7]. CAM can be sorted into two types: biologic therapies such as herbal medicines, vitamin and dietary supplements and non-biologic therapies such as acupuncture, hydrotherapy, massage, and music therapy [4]. A dietary supplement is “the usable forms of the amounts corresponding to high doses of vitamins and minerals and refers to supplements to the nutrients in our diet”. Functional foods are defined as “natural or processed foods that contain known or unknown biologically active compounds that are effective, non-toxic and capable of regulating body functions”. Some examples include ω-3 fatty acids, soluble dietary fibers, probiotics, and prebiotics. Herbal medicines include plants and substances produced from them [8].

Many healthcare professionals tend to fill the existing gaps in the prophylactic and therapeutic measures against COVID 19 using T&CM approaches commonly integrated into conventional medical methods as part of the healthcare system [9]. A wide range of T&CM, including teas, essential oils, vitamins, tinctures, other herbal therapies, etc. have been proposed and investigated from the beginning of the pandemic as the prophylactic and therapeutic measures against COVID-19. In India, early in the pandemic, the Ministry of Health established the AYUSH (Ayurveda, Yoga, Unani, Siddha, and Homeopathy) department that had developed COVID 19 prevention and treatment guidelines using T&CM. The guidelines recommended Yoga and herbal preparations including *Embilica officinalis* (Indian gooseberry), *Ocimum tenuiflorum* (basil), and some commercial products as "immune boosters" [10]. In China the use of traditional Chinese medicine integrated with conventional medicine has limited the spread of the COVID 19 infection effectively [11]. However, in the rest of the world, there is no solid recognition of the safety and effectiveness of T&CM in the treatment of COVID 19 [1,12].

The NIH (National Institute of Health) emphasized that there is inadequate evidence to recommend the use of any vitamin, mineral, herb or other botanical, fatty acid, or other dietary supplement component to prevent or treat COVID 19 [13]. Nevertheless, according to a world-wide infodemiologic study, there was a growth in the sales of dietary supplements by the general population to enhance immunity, prevent or treat COVID 19 [14]. In fact, the sales of dietary supplements raised during the COVID 19 outbreak in majority of the countries [15]. Rachul et al. explored how immune boosting is portrayed on the internet during the first wave (April 2020) COVID 19 pandemic and found that 85.5% of webpages presented the immune boosting as beneficial for preventing COVID 19, with dietary supplements presented as beneficial in 40% of the webpages, most commonly by commercial sites. The top immune boosting methods mentioned in the study were vitamin C (34.8%), diet (34.4%), sleep (34.4%), exercise (30.8%) and zinc (26.9%) [16]. Moreover, it was noted that, despite vaccination and pharmacological treatment for COVID 19 being used successfully, the interest in dietary supplements remains high [17].

Therefore, the WHO established collaboration with academic institutions to identify traditional medicine products that can be tested for clinical efficacy and safety in the treatment of COVID 19 [18].

The Jordanian population was reported to have a high prevalence of herbal medicine use (53.3%), mostly for chronic disease treatments (41.9%) and weight reduction (23.6%) [19]. Even higher frequency (80.8%) of herbal products use was reported in another study from Jordan with the most common reasons for using herbal products being to treat disease, mostly as a self-medication for infertile couples, diabetic, dyslipidaemia, and hypertensive individuals, and to maintain health (44.8%) [20]. A study conducted in herbalist stores in Jordan found that 58% of customers with chronic conditions used herbal medicine; and that a substantial proportion of patients (65.1%) believed in herbal medicine efficacy and 74.5% believed in its safety relative to conventional medicine [21].

Members of the medical team including medical doctors, pharmacists, nurses, dieticians, dentists, etc. are battling COVID 19 on the front line and are particularly at risk of exposure to high viral load. Furthermore, the healthcare professionals and students have the best access to professional information regarding disease treatment and prophylaxis and serve to disseminate such knowledge. The use of T&CM for COVID 19 prevention among healthcare professionals and students of healthcare specialties was addressed in a limited number of studies globally, however, it was not previously assessed in Jordan.

The aim of the study was to assess the prevalence of use of T&CM for the prophylaxis of COVID 19 among the healthcare professionals and students in Jordan, along with the most common types and the factors associated with T&CM use.

## Materials and methods

### Study design and participants

A cross-sectional study of healthcare professionals and students was conducted in Jordan during coronavirus outbreak between June 10, 2021, and August 28, 2021. The researchers use the CHERRIES guidelines for Internet E-Surveys [22] and used Google Forms; a cloud-based survey tool powered by Google™, to build a questionnaire for data collection and to capture responses automatically. The survey was open where different social media platforms, including Facebook®, Facebook Messenger® and WhatsApp Messenger®, were used to advertise and to distribute the questionnaire link. The participants were recruited using a snowball sampling method. They were encouraged to share the link with their colleagues and contacts working in healthcare or studying healthcare specialties.

The study inclusion criteria were:

1. A graduate or a student in one of the following fields: medical doctor, dentist, nurse, physiotherapist, clinical dietician or pharmacist/PharmD.
2. Consented to participate after being informed of the risks and benefits of being involved in the survey.

The exclusion criteria were:

1. Having filled the questionnaire at least once.
2. Pregnant or breast-feeding at the period of the study.

Participants were informed that filling out the questionnaire was voluntary and anonymous and that their responses would only be used for the purpose of the study.

### Study instrument

The questionnaire was developed based on relevant publications [8,23–28] retrieved via extensive literature search of databases including Pubmed and Embase. We have also conducted a search in clinicaltrials.gov [29] for the comprehensive list of ongoing and completed studies of T&CM methods for COVID 19 prevention.

The questionnaire was developed in English with simultaneous translation of each question into Arabic. The questionnaire was then critically revised and face-validated by several academic colleagues specialized in pharmacotherapy and phytotherapy.

The translation process followed the ISPOR guidelines [30]. In brief, two researchers, native speakers of Arabic and fluent in English, performed independent forward questionnaire translation from English into Arabic. This was followed by inspection of the translation by four members of the research group resulting in version 1. Afterwards, a professional translator, a native speaker of English and fluent in Arabic, translated version 1 back to English. The four researchers reviewed this backward translation and provided comments on any items with inaccurate wording or with a change of meaning. We have further asked a person from outside the research group who was not familiar with the original version and the previous backward translation (a healthcare professional who is native speaker in Arabic and fluent in English) to translate into English certain items that were seemingly mistranslated. This was followed by harmonizing the translation by the research group members, resulting in the Arabic version 2. Finally, pilot-testing was conducted on 30 healthcare professionals and students to evaluate the feasibility, comprehensiveness, and verbal clarity of the questionnaire from the participant’s point of view. The participants were asked to comment on words or phrases that were difficult to understand and to provide an alternative, which would be easier and more understandable. Afterwards, the research group reviewed the results of the pilot-testing, followed by making the final changes in the questionnaire. The final version of the questionnaire consisted of three main sections containing 29 items, including screening questions to assess respondents’ eligibility to take the survey, checkbox, dichotomous or matrix questions. Some of dichotomous/checkbox questions were followed by open-ended questions, for example, regarding adverse effects, sources of information. To reduce number and complexity of the questions, certain items were only conditionally displayed based on responses to other items. In more details, the first section included demographic, professional and clinical parameters of the participants such as age, gender, specialty, professional degree, residential place, years of experience, working in contact with patients, including those diagnosed with COVID 19, own history of COVID 19, vaccination status against COVID 19, presence of chronic medical conditions and current medications, including specific questions about the use of aspirin or any anti-infective agents.

The second questionnaire section assessed the frequency and pattern of T&CM use for COVID 19 prophylaxis during the pandemic. The first part of this section assessed whether the participants used vitamins/nutrients for this purpose. A list of vitamin/mineral supplements was provided including vitamin D, vitamin C, zinc, selenium, omega-3 fatty acids or fish oil, multivitamins, probiotics or prebiotics, melatonin, honey, etc. The second part assessed whether participants used non-pharmacological methods of COVID 19 prophylaxis such as steam inhalation, gargling with antiseptics or baby shampoo, nasal washing with saline solution or baby shampoo, keeping hydrated, exercises including breathing exercises, yoga, Tai-chi or Qigong, relaxation techniques (e.g., meditation), extra sleep, etc. The third part assessed whether participants used herbal preparations for COVID 19 prophylaxis such as green tea, garlic, onion, liquorice, anise, ginger, curcuma (turmeric), cloves, cumin, salvia, *Nigella sativa* (black seed), thyme, lemon, peppermint, etc. For the plant selected, participants were asked about the way of preparation, e.g., as a tea, in a raw form, as a food additive, etc. Additionally, participants were asked about plant part used, e.g., fruits, stems, leaves, flowers, roots, bulbs. The concluding question in this section was about the effectiveness of the T&CM method used and the adverse effects, if any.

The third section of the questionnaire contained question regarding the source of information on COVID 19 prevention, such as disease treatment guidelines, scientific publications (result of clinical trials, etc.), social media (Facebook Instagram, Whatsapp groups, etc.), colleagues, professional webinars and TV/radio. Respondents were able to review and change their answers.

### Study sample size

Using a margin of error of 5%, a confidence level of 95%, the population size of healthcare professionals and students approximately of 100000 and a response distribution of 50%, a minimum sample size of 383 is needed for this online survey (Raosoft, Inc.) [31]. To make a sample more representative, the authors decided to include 560 participants.

### Statistical analysis

All data were entered and analysed using SPSS version 21 (SPSS Inc, Chicago, IL).

Categorical variables were expressed as frequencies and percentages, whereas continuous variables were reported as means and standard deviations (SDs). To assess demographic, professional, and clinical parameters linked with T&CM use, the Chi-square and independent sample t-tests were performed. Using odds ratio (OR) values as a measure of association, multiple logistic regression analysis was further utilized to select characteristics that best predicted the T&CM use in the research population. Statistical significance was defined as a P-value of less than 0.05.

### Ethical considerations

The study protocol was approved by the Jordan University’s Deanship of Scientific Research’s Institutional Review Board. Furthermore, this research was carried out in accordance with the Helsinki Declaration. The first page of the Google form contained information on the study’s purpose, goals, and length (not exceeding 20 minutes), as well as participation criteria. Participants were explained that there are no incentives for survey participation. Furthermore, respondents were assured that their participation was entirely voluntary, that their comments would be stored anonymous and used just for research reasons, and that researchers would not be able to identify them. The Google form contained in the beginning a question about consent to participate, and the participants were able to move to the survey questions only in case of providing the consent.

## Results

Among 577 healthcare professionals and students who viewed the survey, 17 declined and 560 agreed to participate, indicating 97.1% response rate. All 560 questionnaires were complete, and none was submitted too soon (less than in 10 minutes), thus, all responses were included in analysis. Demographic and professional characteristics of participants are shown in Table 1. The mean age was 26.83±8.38, with almost half of respondents (44.2%) being in the age category of 21–25 years. Females were more prevalent in our study sample (65.7%) than males (33.2%). Healthcare students and professionals were almost equally represented (53% and 47%, respectively). Among healthcare students, most prevalent (57.6%) were either Pharmacy or PharmD, followed by Medicine (26.0%); one third of all students (33.8%) studying at the fourth year. Most of the healthcare professionals were either PharmD/Clinical Pharmacists (39.8%) or pharmacists (38.3%), followed by medical doctors (17.6%). Among healthcare professionals, 68.4% reported practicing in Jordan at the time of the study, and 72.9% reported practicing at their field of specialization. Most respondents had Jordanian nationality (86.2%), 71.1% being in Amman. More than half of the study participants (57.9%) reported working in contact with COVID 19 patients, and more than two-thirds (78.8%) had been fully vaccinated by the time of the study. Only 14.3% of respondents had previously symptoms of COVID 19 or tested positively for SARS-CoV-2 and 7.9% of them had chronic diseases. Furthermore, 15.9% were receiving medications other than T&CM at the time of the study and 5.2% were receiving aspirin.

**Table 1:** Demographic and professional characteristics of study participants and their association with T&CM use (N = 560).

Participants’ characteristics	N (% within characteristic)	Use of T&CM		P
		Yes, N (% within category)	No, N (% within category)	
Age (y), mean ± sd	26.83±8.38	26.58±7.94	27.29±9.13	0.358*
Age groups (y) 18–20 21–25 26–35 36–45 46–55 >55	86 (15.6) 244 (44.2) 151 (27.4) 46 (8.3) 15 (2.7) 10 (1.8)	49 (57.0) 170 (69.7) 99 (65.6) 24 (52.2) 11 (73.3) 4 (40.0)	37 (43.0) 74 (30.3) 52 (34.4) 22 (47.8) 4 (26.7) 6 (60.0)	**0.047**
Gender Male Female Not reported	183 (33.2) 368 (65.7) 9 (1.1)	109 (59.6) 246 (65.6)	74 (40.4) 123 (33.4)	0.064
Occupation Healthcare student Healthcare professional	297 (53.0) 263 (47.0)	192 (64.6) 167 (63.5)	105 (35.4) 96 (36.5)	0.423
Healthcare students’ specialty Pharmacy/PharmD Medicine Nursing Clinical nutrition Dentistry Rehabilitation	170 (57.6) 77 (26.0) 19 (6.4) 14 (4.7) 10 (3.4) 6 (2.0)	124 (72.9) 37 (48.1) 14 (73.7) 10 (71.4) 5 (50.0) 1 (16.7)	46 (27.1) 40 (51.9) 5 (26.3) 4 (28.6) 5 (50.0) 5 (83.3)	**<0.001**
Healthcare professional specialty PharmD/Clinical pharmacist Pharmacist Medical doctor Others (nurse, clinical nutritionist, dentist)	104 (39.8) 100 (38.3) 46 (17.6) 11 (2.0)	66 (63.5) 62 (62.0) 29 (63.0) 9 (81.8)	38 (36.5) 38 (38.0) 17 (37.0) 2 (18.2)	0.418
Nationality Jordanian Non-Jordanian	483 (86.2) 77 (13.8)	307 (63.6) 51 (66.2)	176 (36.4) 26 (33.8)	0.732
Practicing currently in Jordan Yes No	180 (68.4) 83 (31.6)	119 (66.1) 48 (57.8)	61 (33.9) 35 (42.2)	0.412
Practicing in specialization field Yes No	188 (72.9) 70 (27.1)	117 (62.2) 46 (65.7)	71 (37.8) 24 (34.7)	0.799
For healthcare students, year of the study First Second Third Fourth Fifth Sixth	18 (6.6) 36 (13.1) 58 (21.1) 93 (33.8) 44 (16.0) 26 (9.5)	11 (61.1) 17 (47.2) 37 (63.8) 62 (66.7) 26 (59.1) 22 (84.6)	7 (38.9) 19 (52.8) 21 (36.2) 31 (33.3) 18 (40.9) 4 (15.4)	0.122
Current location Amman Other part of Jordan Outside Jordan	388 (71.1) 126 (23.1) 32 (5.9)	259 (66.8) 78 (61.9) 16 (50)	129 (33.2) 48 (38.1) 16 (50)	0.124
Working in contact with COVID patients Yes No Maybe	171 (30.5) 324 (57.9) 65 (11.7)	122 (71.3) 194 (59.9) 43 (66.2)	49 (28.7) 130 (40.1) 22 (33.8)	**P = 0.015**
Vaccinated against COVID 19 Yes No Maybe	441 (78.8) 115 (20.5) 4 (0.7)	282 (63.9) 75 (65.2) 2 (50)	169 (36.1) 40 (34.8) 2 (50)	0.814
Had symptoms of COVID 19/tested positive for SARS-CoV-2 Yes No Maybe Not answered	80 (14.3) 175 (31.3) 6 (1.1) 299 (53.4)	56 (70.0) 105 (60.0) 6 (100.0) 192 (64.2)	24 (30.0) 70 (40.0) 0 (0.0) 107 (35.8)	0.119
Having chronic illness Yes No Maybe	44 (7.9) 502 (89.6) 14 (2.5)	31 (70.5) 322 (64.1) 6 (42.9)	13 (29.5) 180 (35.9) 8 (57.1)	0.303
Currently receiving medications for other medical problems Yes No Maybe	89 (15.9) 464 (82.9) 7 (1.3)	60 (67.4) 297 (64.0) 2 (28.6)	29 (32.6) 167 (36.0) 5 (71.4)	0.118
Currently receiving aspirin Yes No Maybe	29 (5.2) 516 (92.1) 11 (2.0)	25 (86.2) 323 (62.6) 10 (90.9)	4 (13.8) 193 (37.4) 1 (9.1)	**0.005**

*By independent sample t-test.

P-values in bold indicate statistically significant difference.

Among 560 study respondents, 359 (64.1%) reported using T&CM for COVID 19 prevention. As shown in the Table 2, vitamins and nutrients were consumed by almost half (48.4%) of study participants, while nonpharmacological methods and herbal remedies were consumed by 35.2% and 25.2%, respectively.

**Table 2:** Frequencies of using different T&CM methods for COVID 19 prophylaxis by health professionals.

T&CM method used	Yes, N (%)	No, N (%)	Maybe, N (%)	No answer, N (%)
Vitamins and nutrients	271 (48.4)	252 (45.0)	33 (5.9)	4 (0.7)
Nonpharmacological methods	197 (35.2)	280 (50.0)	74 (13.2)	9 (1.6)
Herbal remedies	141 (25.2)	372 (66.4)	34 (6.1)	13 (2.3)

**Fig 1:**
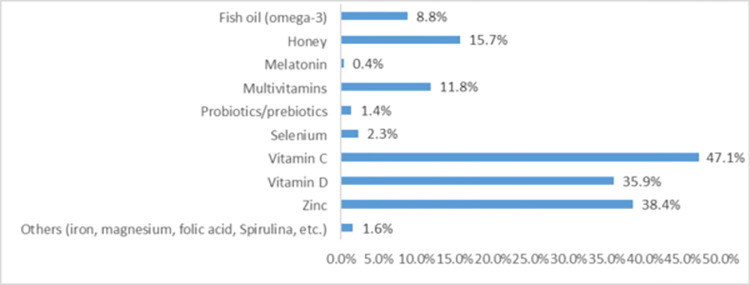
Types of vitamins and nutrients used for COVID 19 prophylaxis (N = 271).

As shown in Fig 1, among vitamins and nutrients used for COVID 19 prophylaxis, the most popular were vitamin C (47.1%), zinc (38.4%), vitamin D (35.9%) and honey (15.7%).

**Fig 2:**
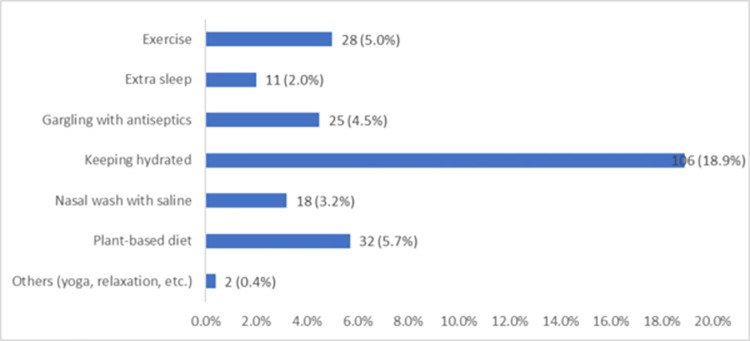
Types of nonpharmacological methods used for COVID 19 prophylaxis (N = 197).

Among nonpharmacological methods used for COVID 19 prophylaxis, the most common included keeping hydrated (18.9%), followed by plant-based diet (5.7%), exercise (5.0%) and gargling with antiseptics (4.5%) (Fig 2).

**Fig 3:**
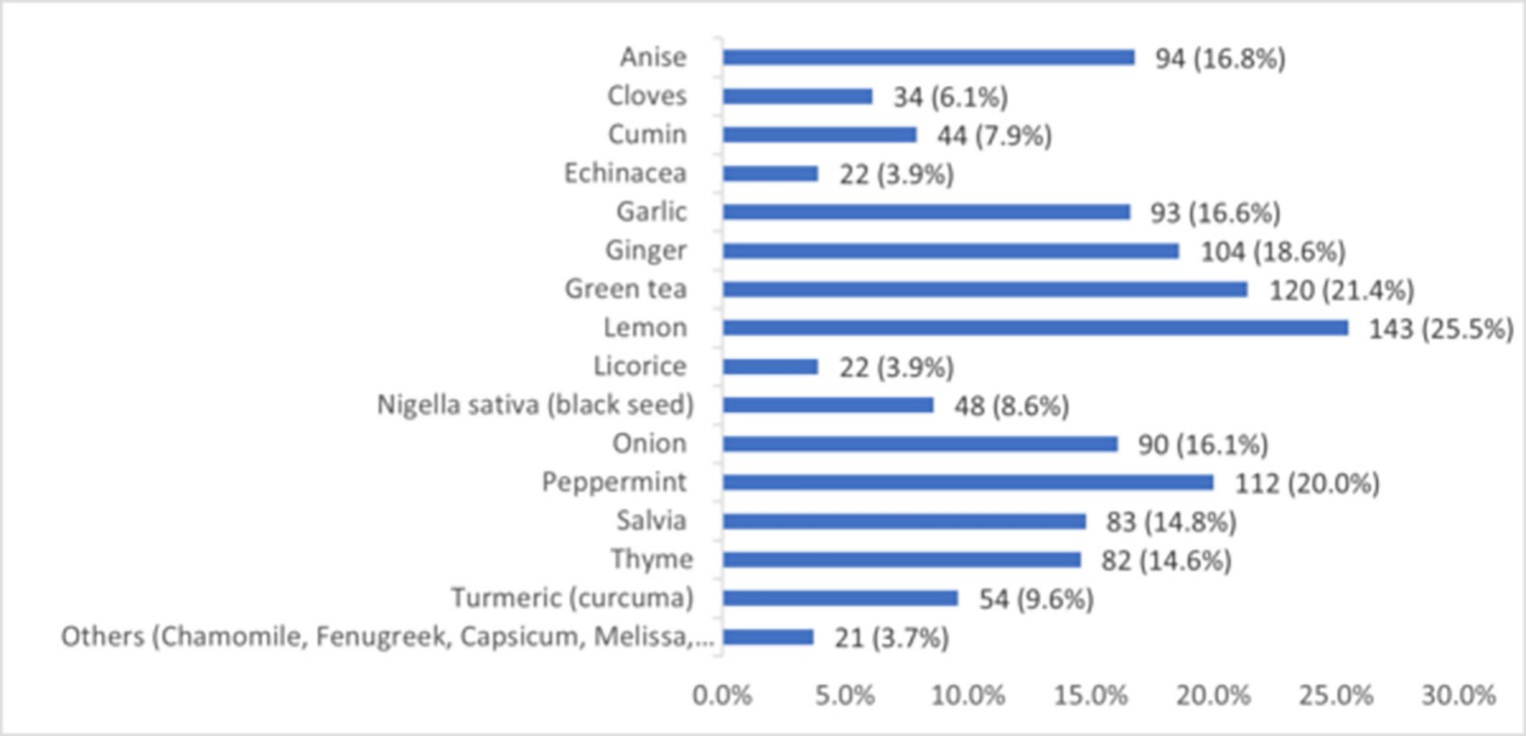
Types of herbal remedies used for COVID 19 prophylaxis (N = 141).

Fig 3 shows that the herbal remedies most frequently used for COVID 19 prophylaxis include lemon (25.5%), green tea (21.4%), peppermint (20.0%), ginger (18.6%), anise (16.8%), garlic (16.6%), onion (16.1%), salvia (14.8%) and thyme (14.6%).

As demonstrated in Table 3, different plant parts were used as herbal remedies for COVID 19 prophylaxis, mostly in the form of teas or food additives.

**Table 3:** Plants’ parts and preparations most used for COVID 19 prophylaxis.

Plant name	Part most used	Most common method of preparation
Green tea	Leaves	Tea
Garlic	Bulbs and leaves	Raw, as a food additive
Onion	Bulbs and leaves	Raw, as a food additive
Liquorice	Roots	Tea
Anise	Seeds	Tea
Ginger	Roots	Tea, in cooking
Turmeric (curcuma)	Root	Tea, in cooking
Cloves	Whole plant	Tea
Cumin	Seeds	Teas, in cooking
Salvia	Leaves	Tea
*Nigella sativa* (black seed)	Seeds	Raw, as a food additive
Thyme	Leaves	Tea and as such
Lemon	Fruits and leaves	Raw, as a food additive, as juice
Peppermint	Leaves	Tea, raw, as a food additive
Echinacea	Different	Tea
Others (Chamomile, Fenugreek, Capsicum, Melissa, Ashwagandha, etc.)	Different	Different

The comparison of demographic and professional characteristics of healthcare professionals and students of healthcare specialties between T&CM users and non-users showed that the age groups below 55 years used T&CM for COVID 19 prophylaxis more commonly than those above 55 years of age (P = 0.047) (Table 1). When comparing students of different specialties, we found that pharmacy, nursing, and clinical nutrition students were more frequently using T&CM prophylaxis compared to medicine and dentistry students (P<0.001). Furthermore, professionals working in contact with COVID 19 patients reported using T&CM prophylaxis against COVID 19 more frequently than those not having contact with such patients (P = 0.015). In addition, respondents who administered aspirin at the time of the study were using T&CM for COVID 19 prophylaxis more frequently than those not receiving aspirin (P = 0.005). As shown in Table 4, the following sources of information were associated with T&CM use for COVID 19 prophylaxis: a pharmacist (P = 0.003), social media (P = 0.024) and a colleague (P = 0.036).

**Table 4:** Sources of information regarding T&CM use for COVID 19 prophylaxis and their association with actual T&CM use.

Information source	Total N (%*)	T&CM use		P
		Yes, N (%^)	No, N (%^)	
Disease treatment guidelines Yes No	213 (38) 347 (62.0)	144 (67.6) 215 (62.0)	69 (32.4) 132 (38.0)	0.176
Scientific publication Yes No	333 (59.5) 227 (40.5)	219 (65.8) 140 (61.7)	114 (34.2) 87 (38.3)	0.322
A pharmacist Yes No	80 (14.3) 480 (85.7)	63 (78.8) 296 (61.7)	17 (21.3) 184 (38.3)	**0.003**
A physician Yes No	95 (17.0) 465 (83.0)	65 (68.4) 294 (63.2)	30 (31.6) 171 (36.8)	0.336
Social media Yes No	184 (32.9) 376 (67.1)	130 (70.7) 229 (60.9)	54 (28.3) 147 (39.1)	**0.024**
A university professor Yes No	89 (15.9) 471 (84.1)	64 (71.9) 295 (62.6)	25 (28.1) 176 (37.4)	0.094
A colleague Yes No	119 (21.3) 441 (78.8)	86 (72.3) 273 (61.9)	33 (27.7) 168 (38.1)	**0.036**
Other Yes No	20 (3.6) 540 (96.4)	15 (75.0) 344 (67.3)	5 (25.0) 196 (36.3)	0.301

*% percentage among all study participants.

^% percentage among category Yes or No.

After entering the above characteristics associated with T&CM use for COVID 19 prophylaxis into logistic regression analysis we found significant associations with working in contact with COVID 19 patients (OR: 1.625 (95% CI 1.047–2.523) (P = 0.03) and having a colleague as a source of information (OR: 1.720 (95% CI 1.026–2.883) (P = 0.04).

**Fig 4:**
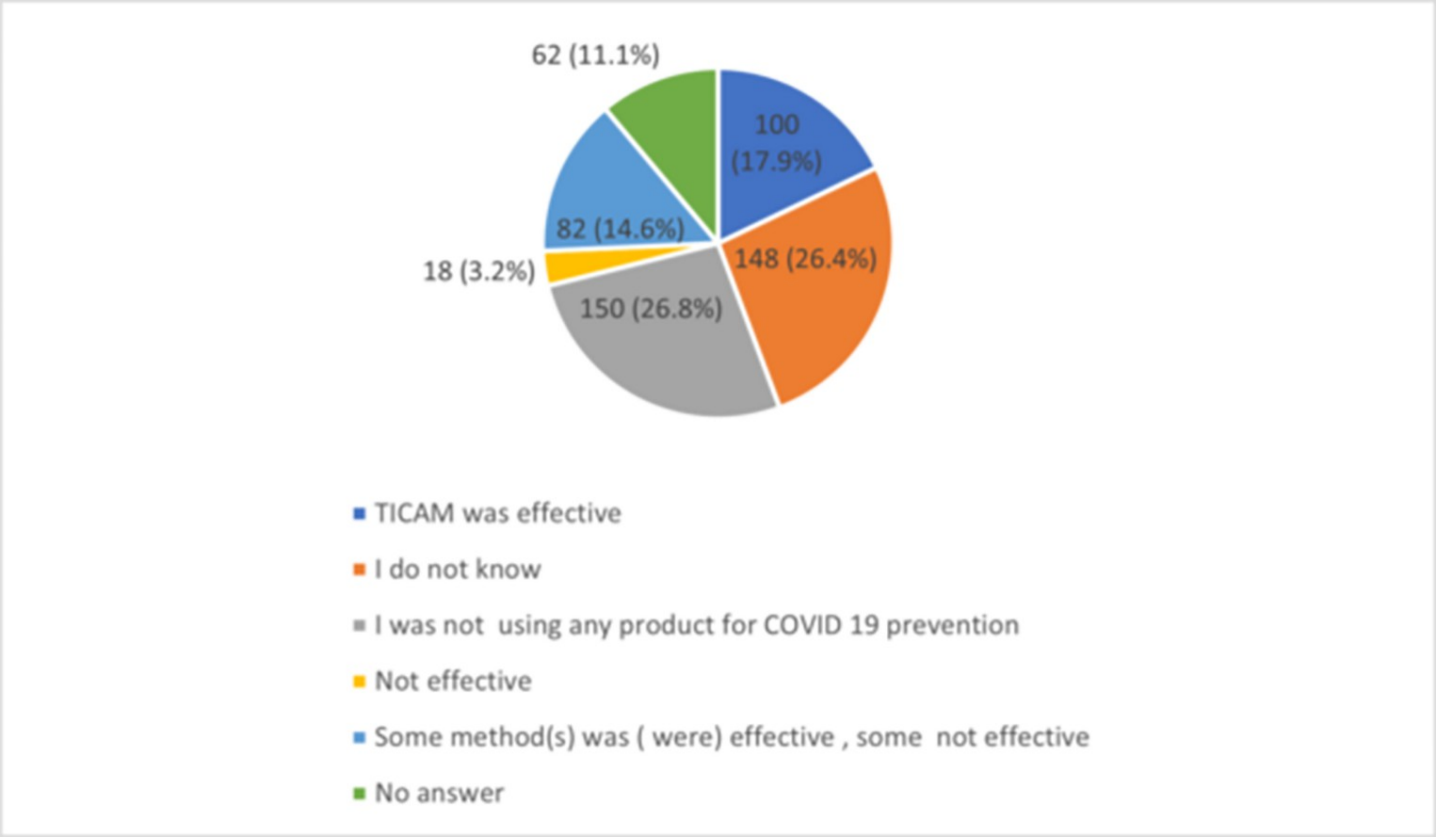
Participants’ opinion regarding T&CM effectiveness.

Among the total sample of participants, only 100 (17.9%) reported that the T&CM method(s) they used for COVID 19 prophylaxis was (were) effective and more than quarter of them (26.1%) did not know whether T&CM was effective (Fig 4).

The most common source of information regarding T&CM use for COVID 19 prophylaxis mentioned by study participants included scientific publications (59.5%), followed by disease treatment guidelines (38.0%) and social media (32.3%) (Table 4).

In our study, total of 17 respondents (8.5%) reported adverse effects and another 17 (8.5%) reported possible adverse effects. As shown in Table 5, the most common adverse effects reported by T&CM users were nausea and/or vomiting, constipation and diarrhea.

**Table 5:** Most common adverse effects experienced by T&CM users.

Method of T&CM (Number of users)	Adverse effects, N (%)					
	Nausea and/or vomiting	Constipation	Diarrhea	Dizziness	Nervousness	Other
Vitamin D (201)	16 (8.0)	1 (0.5)	2 (1.0)	0	0	2 (1.0)
Vitamin C (264)	13 (4.9)	0	1 (0.04)	0	0	3 (1.1)
Zinc (215)	16 (7.4)	2 (1.0)	3 (1.4)	0	0	3 (1.4)
Selenium (13)	5 (38.5)	0	1 (7.7)	0	0	2 (15.4)
Fish oil (omega 3) (49)	8 (16.3)	0	2 (4.1)	0	0	2 (4.1)
Multivitamins (66)	5 (7.6)	0	3 (4.6)	0	1 (1.5)	2 (3.0)
Honey (88)	8 (9.1)	0	2 (2.3)	0	0	3 (3.4)
Echinacea (22)	7 (31.8)	1 (4.5)	0	0	0	2 (9.1)
Green tea (120)	7 (5.8)	0	3 (2.5)	0	0	2 (1.7)
Garlic (93)	3 (3.2)	2 (2.2)	3 (3.2)	0	0	3 (3.2)
Onion (90)	4 (4.4)	1 ((1.1)	3 (3.3)	0	0	3 (3.3)
Liquorice (22)	3 (13.6)	0	2 (9.1)	0	0	2 (9.1)
Anise (94)	3 (3.2)	0	4 (4.3)	0	0	3 (3.2)
Ginger (104)	9 (8.7)	0	2 (1.9)	0	0	3 (2.9)
Turmeric (curcuma) (54)	4 (7.4)	1 (1.9)	3 (5.6)	0	0	3 (5.6)
Cloves (34)	5 (14.7)	0	3 (8.8)	0	0	3 (8.8)
Cumin (44)	3 (6.8)	0	4 (9.1)	0	0	3 (6.8)
Salvia (83)	5 (6.0)	0	0	1 (1.2)	0	3 (3.6)
*Nigella sativa* (black seed) (48)	5 (10.4)	0	1 (2.1)	0	2 (4.2)	2 (4.2)
Thyme (82)	5 (6.1)	1 (1.2)	1 (1.2)	0	0	2 (2.4)
Lemon (143)	6 (4.2)	3 (2.1)	0	0	0	2 (1.4)
Peppermint (112)	8 (7.1)	0	1 (0.9)	0	0	2 (1.8)

## Discussion

With the rapid spread of COVID 19, the disease was life-threatening and caused psychological distress and anxiety for both healthcare professionals and the public in many parts of the world. The situation was exacerbated by inadequate medical infrastructure, essential medical equipment in medical facilities, and inadequate health care workers, encouraging self-medication to alleviate the discomfort and anxiety caused by burnout [32]. Due to the collective efforts of medical professionals, researchers, and decision makers, in most parts of the world, the COVID 19 pandemic is now brought under effective control. However, the battle against COVID 19 is ongoing in and studies are needed to assess health care professionals’ knowledge, attitudes, and practices in T&CM use for COVID 19 prophylaxis.

Several studies investigated the prevalence and patterns of T&CM use for COVID 19 prevention, with only few of the studies focused on the T&CM use among healthcare professionals and students.

As for the public, a cross-sectional study in Togo that included participants from five sectors, including healthcare found the overall predominance of self-medication to prevent COVID 19 was 34.2% [26]. An electronic survey conducted in Ghana reported that 69.1% of T&CM users intended it for the prevention of COVID 19 [33]. Furthermore, in a recent nested case-control study of home-based remedies to prevent COVID 19-associated risk of infection, admission, severe disease, and death conducted in Ghana, almost every third person presenting for COVID 19 test used some form of home-based remedy to prevent the disease [34]. In a survey of 782 Iranian residents 50–66% of them used T&CMs to prevent the disease transmission or to reduce anxiety caused by the COVID 19 pandemic; the most used T&CMs were dietary supplements (61.3%), prayer (57.9%), and herbal medicines (48.8%) [4]. In a cross-sectional study from Turkey, the adults widely used T&CM methods (70.5%) during the COVID 19 pandemic, and they were more oriented toward the use of herbal treatment (35.5%) [35]. Lower prevalence of T&CM use for COVID 19 prevention was observed in Europe: an online survey conducted during the first wave of COVID 19 in Netherlands (May 2020) showed that 68.0% of the participants used complementary medicine, mainly to improve general wellbeing (61.6%), and only in 10% of participants for prevention and/or treatment of COVID 19 [36]. The anonymous questionnaire survey among patients of a Hungarian university hospital and a city hospital waiting for elective surgery found that only 10% of respondents using such therapies to prevent coronavirus infection [37]. Similarly, in a population-based cross-sectional study conducted in spring 2020 in three European countries (Norway, Sweden, and Netherlands), a very low proportion of responders used self-management strategies to prevent or treat COVID 19 (3.4% and 0.2% respectively); with vitamins and minerals being the most used for prevention of COVID 19 (2.8%) [38].

Regarding healthcare workers, the highest prevalence of T&CM use for COVID 19 prevention among dieticians was reported in Turkey in the beginning of the pandemic, where 94.5% of the participants used dietary supplements, 46.1% used herbal medicines and 34.9% used functional foods to avoid COVID 19 during the pandemic [8]. In another study from Turkey among health care workers, mostly (65%) nurses, 45.5% of the participants used T&CM methods for COVID 19 during the last month and 48.7% of the health care personnel stated that they used complementary and alternative medicine (CAM) methods to strengthen their immune system. Additionally, there was a positive association between the CAM use and life satisfaction [28]. A study from Nigeria sought to assess the knowledge, attitude, and practice of medical students on complementary and alternative medicine in the management of COVID 19. It was found that students have good knowledge (75.3%) and a positive attitude (74.7%) towards CAM modalities as adjunct management for COVID 19, however, their practices do not reflect wide acceptability [39]. In our study, the prevalence of T&CM use by healthcare professionals and students was high (64.1%), including vitamins and nutrients (48.4%), nonpharmacological methods (35.2%) and herbal remedies (25.2%). The differences in T&CM use for COVID 19 prevention among the studies may be due to the inclusion of healthcare professionals from various fields; in our study the most prevalent respondents were PharmDs, clinical pharmacists or pharmacists, additionally, students from the above specialties comprised roughly half of the study sample. Furthermore, in the studies conducted in the beginning of COVID 19 outbreak the medical professionals could not possess sufficient knowledge regarding the effectiveness and safety of T&CM in preventing COVID 19. Expectedly, the traditional and cultural differences among the countries also have the impact on the prevalence and patterns of T&CM use.

As for the patterns of specific T&CM methods used for COVID 19 prophylaxis, they also vary widely across different cultures. In a Turkish survey among dieticians during the COVID 19 outbreak the most used dietary supplement was fish oil (81.9%), among functional foods the most prevalent were vegetables and fruits (80.5%) and among herbal medicine the most widely used was cinnamon (63.5%) [8]. As for the public, an electronic survey conducted in Ghana reported that vitamin supplements (88.1%), spiritual healing/prayer (23.3%), mineral supplements (22.3%), herbal products (22.2%), and diet therapy (19.4%) were the main types of T&CM used [33]. In a study conducted in Bangladesh, almost half of the participants reported using ginger and honey [40]. In Togo, vitamin C (27.6%) and traditional medicine were the most widely utilized products (10.2%) [26]. In Nepalese research of natural herbs used to prevent COVID 19, ginger was the most frequently cited, followed by curcumin (frequency of citations 0.398 and 0.341, respectively) [41]. A global Google trends analysis supported by PLifeCOVID 19 online studies of dietary supplements during COVID 19 outbreak showed that the Middle Eastern countries tended to search vitamins, vitamin D, zinc, onion, raspberry, and *Nigella sativa* [15]. In Saudi Arabia, honey, lemon, and ginger were among the most utilized natural products as a preventative precaution against COVID 19 [17]. Furthermore, two previous cross-sectional surveys conducted in Jordan published some data regarding prophylactic use of T&CM during COVID 19 pandemic. An online survey conducted between 26 March and 16 April 2021 assessed self-medication use to prevent or treat COVID 19; notably, 40% of participants worked or studied in medical field. The most used natural products to self-medicate were vitamin C (57.6%), zinc (44.8%) and vitamin D (32.5%) [42]. The other survey was conducted on May 19th-July 29th, 2021, which included 15% of participants working in medical sector. More than 70% of the respondents used vitamins C and D, while the most used natural products were citrus fruits (78.8%), honey (63.0%), ginger (53.1%), cinnamon (35.0%), star anise (32.1%) and clove (27.5%) [17]. The T&CMs most frequently used by healthcare professionals and students for COVID 19 prophylaxis in our study generally match the above data reported for general population and dieticians, and, among herbal remedies, include lemon (25.5%), green tea (21.4%), peppermint (20.0%), ginger (18.6%), anise (16.8%), garlic (16.6%), onion (16.1%), salvia (14.8%) and thyme (14.6%), while among vitamins and nutrients, the most popular were vitamin C (47.1%), zinc (38.4%), vitamin D (35.9%) and honey (15.7%). This is not surprising, as in a previous study conducted in Jordan, the use of herbal medicines was widely advocated by pharmacists for the prevention of COVID 19 symptoms [43].

There are possibly two reasons explaining the choice of these T&CM methods of COVID 19 prophylaxis by healthcare professional and students in Jordan. First, by the time of our study initiation, there were several publications that contained data on immune-boosting, anti-inflammatory, antioxidant, antimicrobial and antiviral properties of several nutrients and herbal medicines, including vitamins D and C, zinc, selenium, garlic, ginger, turmeric, bitter substances, herbs and spices, herbal teas, and Nigella sativa [9]. Subsequently, products such as vitamins D and C, zinc, probiotics, curcumin, and many others have been extensively studied in the relation to prevention and treatment of SARS-CoV-2 in clinical trials; though, with no solid evidence [15]. Secondly, most of the herbal products used for COVID 19 prophylaxis in our study are already described to be used very commonly (Salvia (sage) and Nigella sativa (black seed)) or commonly (Zingiber officinalis (ginger), Cinnamomum ceylanicum (cinnamon), Thymus vulgaris (thyme)) for the treatment of common cold in Jordan [44].

Among nonpharmacological methods, the most reported in our study were keeping hydrated (18.9%), plant-based diet (5.7%), exercise (5.0%) and gargling with antiseptics (4.5%). In the Netherlands, an online survey was used to study lifestyle-related changes among randomly selected adults during COVID 19 outbreak. The authors observed changes to a healthier lifestyle in 19.3% of the population, mainly due to a change in diet habits, physical activity, and relaxation. Change to a healthier lifestyle was positively associated with ’CAM use’ (OR: 2.04, 95% C.I. 1.38–3.02), among the other variables [45]. Of note, maintaining a balanced diet, staying well hydrated, exercising regularly, and sleeping well was recommended by the WHO as a general measure to stay in good health [46].

Factors associated with T&CM use for COVID 19 prophylaxis were previously reported for the general population. In an electronic survey conducted in Ghana the predictors of CAM use were the age, gender, participants’ perceptions of consequences, identity, and concerns about COVID 19 [33]. In an Iranian survey, factors associated with CAM use were gender, having children, place of residence, COVID 19 status, and source of information about CAMs [4]. In an online survey conducted during the first wave of COVID 19 in Netherlands (May 2020) complementary medicine was used for prevention and/or treatment of COVID 19 most commonly by women and highly educated individuals [36]. In our study, among healthcare professionals and students, T&CM use for COVID 19 prevention was significant associated only with two factors, namely, working in contact with COVID 19 patients and having a colleague as a source of information. Furthermore, a recent systematic review and meta-analysis that looked into the determinants of traditional, complementary and integrative medicine (TCIM) interventions use for COVID-19, concluded that, for underdeveloped countries, TCIM interventions tended to be accepted as a panacea due to shortage of medical resources and restricted access to medical institutions, while the high prevalence of TCIM usage in Western countries might be due to dissatisfaction with the quality of conventional healthcare services [47].

Several studies investigated efficacy and safety of T&CM methods in the treatment of COVID 19 patients, including Chinese patent medicines Jing-Si Herbal Tea [48] and Lianhua Qingwen [49], flavonoids [50], and Ayurcov, a formulation made of ingredients mentioned in Ayurveda [51]. In addition, a retrospective study in Egypt included COVID 19 patients and their contacts who used TaibUVID nutritional supplement from prophetic medicine (*Nigella sativa*, chamomile, and natural honey). Along with therapeutic benefits of TaibUVID, it helped COVID 19 contacts’ prophylaxis where 70% of COVID 19 contacts (n = 14) on regular TaibUVID intake did not get SARS-COV2 infection. Of note, COVID 19 contacts were either mainly physicians (40%, n = 8) dealing with COVID 19 patients daily or members of physicians’ families (45%) [52]. An online survey conducted during the first wave of COVID 19 in Netherlands (May 2020) reported that the participants who used complementary medicine for prevention and/or treatment of COVID 19 seemed to benefit of it [35]. Furthermore, in a recent study that used supervised machine learning approach for sentiment and emotion analysis of Twitter content related to complementary, alternative, and integrative medicine use in relation to COVID 19, most of tweet’s subset was positive, and the authors interpreted them as public support for T&CM [1]. On the contrary, among our study participants, only 17.9% reported that the T&CM method(s) they used for COVID 19 prophylaxis was (were) effective and more than quarter of them were not sure of T&CM effectiveness in T&CM prevention. Regarding the most recent data on T&CM, a comprehensive overview of systematic reviews that summarized the evidence for CAM interventions in the treatment of COVID-19 patients, including vitamin D, herbs, physical exercise and traditional Chinese herbal medications (TCM) found that, among all interventions, only TCM decreased the rate of disease progression (relative risk (RR) 0.30, 95% confidence intervals (CI) [0.20, 0.44]), time to the resolution of fever (standard mean difference (SMD) - 0.98, 95% CI [-1.78, -0.17]) and rate of progression to severe COVID-19 cases (RR 0.34, 95% CI [0.18, 0.65]) [53].Although T&CM methods are generally thought of as benign because of their easy accessibility, they are not necessarily free from adverse effects [54]. In the COVID A to Z Study [55] more adverse effects (nausea, diarrhea, and stomach cramps) were reported in the dietary supplement groups than in the usual care group. More alarmingly, there were reports of acute hepatitis in six patients during the study period of 4 months in the COVID 19 pandemic due to *Tinospora cordifolia*, an immune boosting plant frequently used in India’s traditional system of Ayurveda for COVID 19 prophylaxis [56]. Furthermore, in a study conducted in Ghana, steam inhalation and herbal baths increased the risk of infection, while physical exercise and dietary changes protected against COVID 19 infection and hospital admission [34]. The Egyptian study reported that TaibUVID was tolerable, and majority (81.25% of COVID 19 patients) did not report side effects, while 18.25% reported mild diarrhea, sweating and hyperglycemia (not confirmed to be due to TaibUVID supplements) [52]. In our study, 8.5% respondents reported adverse effects and another 8.5% reported possible adverse effects, with the most common being nausea and/or vomiting, constipation and diarrhea. Notably, in two studies conducted in Jordan before COVID 19 outbreak the adverse effects were reported by approximately a quarter of herbal product users [19], most commonly, vomiting and nausea (9.3%) [20].

These results suggest the need for appropriate public health policy on COVID 19 and T&CM use in addition to long-term clinical trials to ensure the efficacy and safety of T&CM, the latter particularly important for patients with comorbidities who are at risk of adverse effect or drug interaction with their ongoing medications [23,33].

Regarding the information sources for the T&CM use for COVID 19 prophylaxis, two previous Jordanian studies conducted on general population during COVID 19 outbreak, reported that pharmacists were the second most common source of participants’ information about drugs to prevent or treat COVID 19. In the first study, pharmacists (43.4%) ranked second after newspapers (44.0%) [42], while in the second study, pharmacists (54.1%) followed family and friends (55.4%) [17]. Furthermore, in a study that focused on a role of pharmacists in COVID 19 disease conducted in the beginning of COVID 19 outbreak (March 2020) in Jordan, the most common sources of information used by the respondents (80.6%) were professional websites including the World Health Organization (WHO), Centers for Disease Control and Prevention (CDC), or the International Pharmaceutical Federation (FIP), followed by the Jordanian television and official sites (61.2%) and physicians (41.6%) [43]. Notably, in our study, where most participants were pharmacists or PharmD, the most common sources of information regarding T&CM use for COVID 19 prophylaxis were scientific publications (59.5%), followed by disease treatment guidelines (38.0%) and social media (32.3%). It is worth mentioning data from Finland that individuals with less trust in sources of information regarding COVID 19 and more endorsement of CAM were more unwilling to take a COVID 19 vaccine [27]. All this underscores the importance of using professional medical sources of information by the members of healthcare team as they are used as reference points by public.

### Study strengths and limitations

Among the strengths of the online survey, we were able of reaching the healthcare professionals and students from different specialties and from different geographical areas in Jordan and, to collect data over short period of time. Another study strength is its comprehensiveness: we not only focused on T&CM methods used for COVID 19 prevention specifically among healthcare professionals and students, but also investigated a wide range of T&CM approaches, their specific types, factors associated with T&CM use, participants’ opinion regarding efficacy and safety as well as the sources of information.

One of the limitations of this study was participant self-selection, as the survey was conducted online utilizing social media platforms with snowball method. Thus, only respondents who use the Internet and social media participated in the study, however, Internet services are already widely used by healthcare professionals and students, especially in COVID 19 era. Another limitation is the representativeness of the sample to the healthcare professionals/students of Jordan: females were slightly more prevalent than males, the most prevalent age category was 21–25 years, and the most prevalent category of health care professionals and students were pharmacists/PharmDs/clinical pharmacists. In addition, most of the participants were from Amman, the capital of Jordan. Furthermore, we were able to detect the response rate only from the number of participants who have checked the box “I agree to participate”, but not by detecting the unique visitors using cookies or the IP address or both. Also, due to the open nature of the survey, we were not able to prevent duplicate entries from the same user. Therefore, these limitations open an avenue for future studies to observe the T&CM use for COVID 19 prophylaxis.

## Conclusions

The prevalence of T&CM use for COVID 19 prevention among healthcare professionals and students in Jordan is high, with a significant proportion of participants reporting adverse effects. There is an urgent need for further research toward efficacy and safety of T&CM in COVID 19 prophylaxis as well as development of appropriate public health policy on this issue specific to each country.

## Supporting information

S1 File (CSV)
